# Right Atrioventricular Valvular Dysplasia in a New Zealand White Rabbit

**DOI:** 10.1155/2021/6674024

**Published:** 2021-02-04

**Authors:** Scott D. Reed, Melanie E. Blaisdell

**Affiliations:** Biocompatibility Department, NAMSA, Northwood, Ohio, USA

## Abstract

A sixteen-week-old, male New Zealand White rabbit was euthanized following an acute onset of respiratory distress and cyanosis. On necropsy, the rabbit had marked right atrioventricular eccentric hypertrophy, absence or rudimentary presence of the septal leaflet of the right atrioventricular valve, focally extensive left ventricular infarction, diffuse hepatic chronic passive congestion, and diffuse pulmonary edema. To our knowledge, right atrioventricular valvular hypoplasia, dysplasia, or aplasia has not been previously described in rabbits.

## 1. Introduction

Congenital heart defects are seldom reported in rabbits. The few cases described in the literature include ventricular septal defect with aortic valve insufficiency [1], atrial septal defect [2], and partial atrioventricular septal defect [3]. No publications referring to right atrioventricular valvular (RAV) hypoplasia, dysplasia, or aplasia in rabbits have been published. In contrast, congenital lesions in dogs, cats, and humans are well documented and RAV insufficiency and related congenital defects have been well documented in these species [4–12]. In humans, the most commonly reported defect of the RAV is Ebstein's anomaly (EA) [5]. EA is a malformation of the RAV and right ventricle characterized by (1) adherence of the septal and posterior leaflets to the underlying myocardium; (2) apical displacement of the functional annulus; (3) dilation of the “atrialized” portion of the right ventricle, with various degrees of hypertrophy and thinning of the wall; (4) redundancy, fenestrations, and tethering of the anterior leaflet; and (5) dilation of the right atrioventricular junction. Although EA includes all aforementioned criteria, various RAV anomalies that do not include all criteria have also been described [5, 6, 9–11]. Most of the nonhuman species with described RAV anomalies do not fit all criteria for EA, but the dog has been suggested as a model of the syndrome, and both humans and dogs have had the anomaly mapped to a specific genetic defect [4]. Similarly, it is likely that most RAV anomalies are genetic in other species; however, developmental anomalies of the RAV have also been associated with prenatal exposure to cyclooxygenase inhibitors [6] and RAV insufficiency secondary to trauma in a kitten has been described [7].

It should be noted that rabbits are unique in that they normally have a bicuspid right atrioventricular valve; therefore, RAV is the anatomic term used throughout this manuscript to include both the bicuspid valve in rabbits and the tricuspid valve in other species. In this report, the gross pathology of the first known reported case of right atrioventricular valvular dysplasia in rabbits is described and depicted.

## 2. Materials and Methods

A twelve-week-old, male New Zealand White rabbit was received from Robinson Services Incorporated (RSI), 158 Kayla Trail, Post Office Box 1057, Mocksville, NC 27028, and underwent quarantine and acclimation. After five days of quarantine and acclimation, the animal was maintained in a vivarium with other stock rabbits prior to being placed onto the study. Four weeks following receipt, the animal started exhibiting clinical signs of acute respiratory distress and cyanosis. Based on the animal's deteriorating condition and distress, euthanasia was performed by intravenous injection of a pentobarbital-based euthanasia solution. A full necropsy was performed, and findings are the subject of this manuscript.

Animal husbandry, euthanasia, and all procedures were in compliance with all applicable principles set forth in the National Institutes of Health Guide for the Care and Use of Laboratory Animals (8^th^ Edition, revised 2011).

## 3. Results

**Figure 1:**
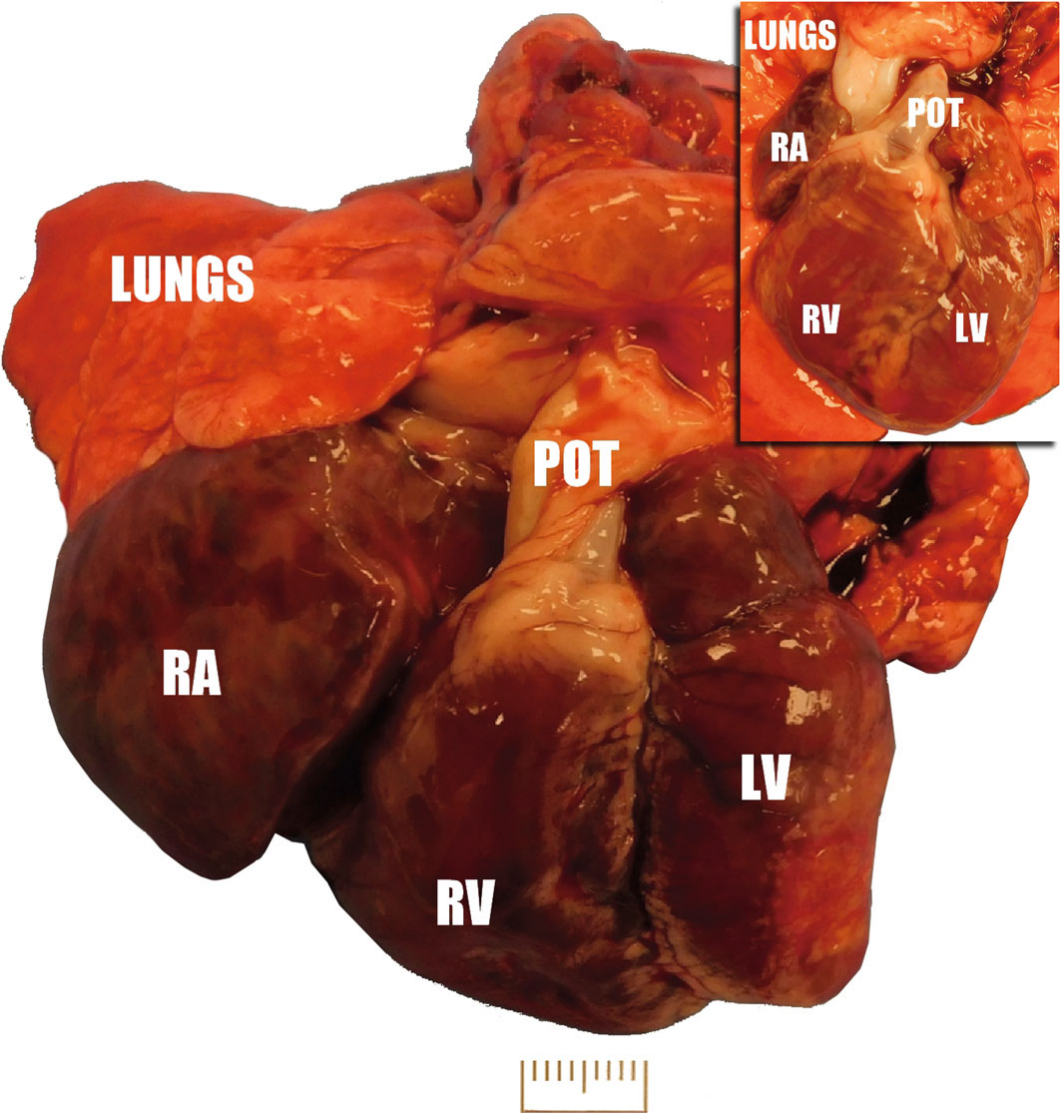
Gross image of the heart and lungs—caudal view of the heart illustrating a markedly enlarged right atrium (RA) and an enlarged right ventricle (RV). The left ventricle (LV) was slightly smaller than the right ventricle. Lungs were partially compressed by the enlarged heart, were moist and exuded fluid on sectioning, and had a rubbery texture. The pulmonary artery and entire pulmonary outflow tract (POT) were also enlarged and dilated. Inset: an image of an age-matched rabbit is provided for comparison. Scale bar = 1 cm.

**Figure 2:**
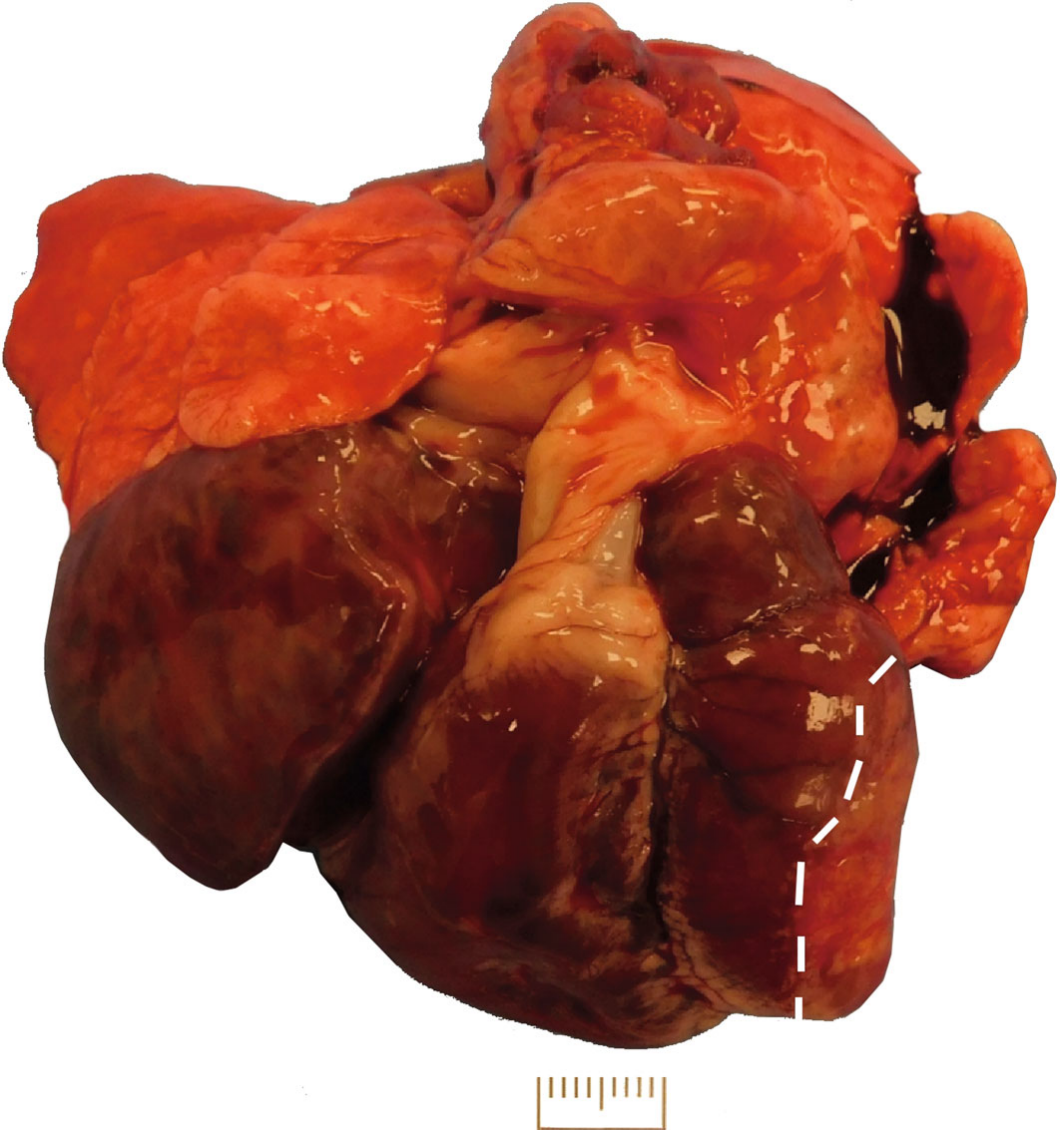
Gross image of the heart and lungs—caudal view of the heart. The left ventricle had a focally extensive depressed firm white area encompassing approximately half of the ventricular free wall (outlined in white dashes). Scale bar = 1 cm.

On necropsy, diffusely wet firm rubbery lungs were partially compressed by a markedly enlarged heart. Heart enlargement was characterized by marked right-sided heart enlargement composed of a right atrium that was more than doubled in size and a right ventricle with eccentric hypertrophy and enlargement by approximately 80-100% (Figure 1). The left ventricular free wall was characterized by partially thinned, white, fibrous tissue comprising approximately 30% of the ventricle consistent with a chronic infarct (Figure 2).

**Figure 3:**
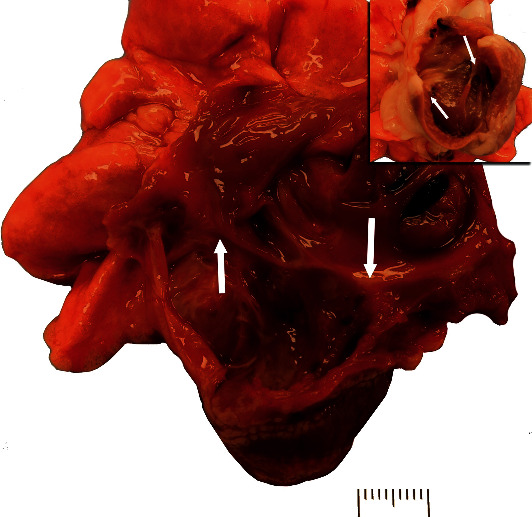
Gross image of the right atrial and ventricular lumina. The lumina of the right atrium and ventricle were markedly dilated as was the atrioventricular annulus. A fibrous ridge characterizes the annulus between the atrium and ventricle, and a single valve leaflet (free wall) is seen with a hypoplastic cusp (right arrow). The location of where the septal leaflet was expected (left arrow) has no leaflet (cusp aplasia) and no associated chordae tendineae. The chordae tendineae associated with the free wall leaflet were more characteristic of a thickened fenestrated membrane than individual well-developed chordae tendineae. The right wall thickness was within normal limits, but overall right heart mass was increased (eccentric hypertrophy). Inset: an image of an age-matched rabbit is provided for comparison. Scale bar = 1 cm.

Upon opening the heart, the right atrioventricular annulus was markedly dilated and no definitive septal cusp was identified (chordae tendineae truncated at the fibrous ridge forming the right atrioventricular annulus); the free wall cusp appeared smaller than normal and had a decreased number of chordae tendineae attaching to it (Figure 3). Systemically, the only other notable finding was a liver with minimal enlargement, an accentuated lobular pattern, and rounded margins.

## 4. Discussion

We describe a rabbit with decompensated congestive heart failure resulting from developmental anomalies in the RAV. On gross necropsy, the rabbit had moist, rubbery, partially compressed but not collapsed lungs, a slightly enlarged liver with an accentuated lobular pattern and rounded margins, and a markedly enlarged heart dominated by right atrial and ventricular enlargement. The heart had malformation of the RAV characterized by a hypoplastic free wall valve leaflet, malformed chordae tendineae associated with the free wall leaflet, aplasia of the septal wall leaflet, and associated chordae tendineae, and a markedly dilated annulus. Associated with the RAV anomaly was a markedly dilated right atrium and right-sided eccentric myocardial hypertrophy. Additionally, an incidental finding of left ventricular free wall infarction was seen.

This is the first case of RAV malformation published in the rabbit. Clinical as well as pathologic cardiac and systemic changes were consistent with RAV insufficiency. Liver findings were consistent with chronic passive congestion which is an expected finding with decompensated right heart failure. Pulmonary changes were consistent with pulmonary edema which is an expected finding with decompensated congestive left heart failure. Both the hepatic and pulmonary findings were characteristic of biventricular heart failure as were the antemortem clinical findings.

Non-RAV anomaly cardiac changes in the right heart were consistent with RAV insufficiency as well. Right atrial dilation is an expected outcome of chronic RAV regurgitation. Right-sided myocardial eccentric hypertrophy is an expected compensatory mechanism associated with RAV insufficiency. The infarct of the left ventricular free wall is not completely unexpected given the propensity of animals with valvular incompetence to have reflux rheostatic alterations resulting in turbulence and increased susceptibility to thromboemboli. It is uncertain if the left heart failure alterations were a consequence of infarction of the left ventricle or of preload decreases secondary to right heart failure.

Given the unique structure of the rabbit RAV (bicuspid), it is not surprising that many of the RAV anomaly changes in this species are different than those described in other species and the consequences of malformation of a single leaflet are much more consequential. Criteria associate with human EA were not met, but some of the changes seen in that anomaly were present in this case. Given the lack of any history of any gestational or postgestational xenobiotic use and the lack of a history of early trauma, this rabbit's RAV anomalies were thought to be genetic in this rabbit and other changes were a consequence of RAV insufficiency.

## Abbreviations

EA: Ebstein's anomaly
RAV: Right atrioventricular valve.

## References

[1] Voros K., Seehusen F., Hungerbuhler S., Meyer-Lindenberg A., von der Hoeh N. (2011). Ventricular septal defect with aortic valve insufficiency in a New Zealand White rabbit. *Journal of the American Animal Hospital Association*.

[2] Nakata M., Miwa Y., Chambers J. K., Saito T., Uchida K. (2018). Ostium secundum type of atrial septal defect in a rabbit. *Journal of Veterinary Medical Science*.

[3] di Girolamo N., Palmieri C., Baron Toaldo M. (2018). First description of partial atrioventricular septal defect in a rabbit. *Journal of Exotic Pet Medicine*.

[4] Andelfinger G., Wright K. N., Lee H. S., Siemens L. M., Benson D. W. (2003). Canine tricuspid valve malformation, a model of human Ebstein anomaly, maps to dog chromosome 9. *Journal of Medical Genetics*.

[5] Attenhofer Jost C. H., Connolly H. M., Dearani J. A., Edwards W. D., Danielson G. K. (2007). Ebstein’s anomaly. *Circulation*.

[6] Burdan F., Szumilo J., Dudka J., Korobowicz A., Klepacz R. (2006). Congenital ventricular septal defects and prenatal exposure to cyclooxygenase inhibitors. *Brazilian Journal of Medical and Biological Research*.

[7] Closa J. M., Font A. (1999). Traumatic tricuspid insufficiency in a kitten. *Journal of the American Animal Hospital Association*.

[8] Famula T. R., Siemens L. M., Davidson A. P., Packard M. (2002). Evaluation of the genetic basis of tricuspid valve dysplasia in Labrador Retrievers. *American Journal of Veterinary Research*.

[9] Formigari R., Francalanci P., Gallo P. (1993). Pathology of atrioventricular valve dysplasia. *Cardiovascular Pathology*.

[10] Kobza R., Kurz D. J., Oechslin E. N. (2004). Aberrant tendinous chords with tethering of the tricuspid leaflets: a congenital anomaly causing severe tricuspid regurgitation. *Heart*.

[11] Lang D., Oberhoffer R., Cook A. (1991). Pathologic spectrum of malformations of the tricuspid valve in prenatal and neonatal life. *Journal of the American College of Cardiology*.

[12] Robinson N. A., Armíen A. G. (2010). Tubular hypoplasia of the aorta and right atrioventricular valve dysplasia in a bulldog. *Journal of Veterinary Diagnostic Investigation*.

